# Sensor Failure Tolerable Machine Learning-Based Food Quality Prediction Model

**DOI:** 10.3390/s20113173

**Published:** 2020-06-03

**Authors:** Aydin Kaya, Ali Seydi Keçeli, Cagatay Catal, Bedir Tekinerdogan

**Affiliations:** 1 Department of Computer Engineering, Cankaya University, Ankara 06790, Turkey; 2 Department of Software Engineering, Cankaya University, Ankara 06790, Turkey; aliseydi@cankaya.edu.tr; 3 Department of Computer Engineering, Bahcesehir University, Istanbul 34353, Turkey; cagatay.catal@eng.bau.edu.tr; 4 Information Technology Group, Wageningen University & Research, 6706 KN Wageningen, The Netherlands

**Keywords:** classifier, single plurality voting system, ensemble classifier, machine learning, beef cut quality prediction

## Abstract

For the agricultural food production sector, the control and assessment of food quality is an essential issue, which has a direct impact on both human health and the economic value of the product. One of the fundamental properties from which the quality of the food can be derived is the smell of the product. A significant trend in this context is machine olfaction or the automated simulation of the sense of smell using a so-called electronic nose or e-nose. Hereby, many sensors are used to detect compounds, which define the odors and herewith the quality of the product. The proper assessment of the food quality is based on the correct functioning of the adopted sensors. Unfortunately, sensors may fail to provide the correct measures due to, for example, physical aging or environmental factors. To tolerate this problem, various approaches have been applied, often focusing on correcting the input data from the failed sensor. In this study, we adopt an alternative approach and propose machine learning-based failure tolerance that ignores failed sensors. To tolerate for the failed sensor and to keep the overall prediction accuracy acceptable, a Single Plurality Voting System (SPVS) classification approach is used. Hereby, single classifiers are trained by each feature and based on the outcome of these classifiers, and a composed classifier is built. To build our SPVS-based technique, K-Nearest Neighbor (kNN), Decision Tree, and Linear Discriminant Analysis (LDA) classifiers are applied as the base classifiers. Our proposed approach has a clear advantage over traditional machine learning models since it can tolerate the sensor failure or other types of failures by ignoring and thus enhance the assessment of food quality. To illustrate our approach, we use the case study of beef cut quality assessment. The experiments showed promising results for beef cut quality prediction in particular, and food quality assessment in general.

## 1. Introduction

For the agricultural food production sector, the control and assessment of food quality is an essential issue, which has a direct impact on both human health and the economic value of the product. Food quality defines the essential and distinguishing characteristics of food that is acceptable to consumers. Quality characteristics include external factors such as appearance, texture, and flavor, or internal factors such as chemical, physical, or microbial properties. One of the crucial properties from which the quality of the food can be derived is the smell of the product, which is the major contributing perception of the food aroma and flavor. The related term olfaction is defined as the perception of smell and can usually be done by human experts. However, a significant trend in this context is machine olfaction [1], or the automated simulation of the sense of smell using a so-called electronic nose or e-nose. Machine olfaction has been applied in different fields for several purposes, such as food quality control [2], freshness evaluation of meat [3], detection of fresh vegetable freezing time [4], illegal substance detection [5], diagnosis of infections [6] and diagnosis of diseases [7,8]. Machine olfaction involves the use of automated systems, or electronic nose (e-nose) to analyze air-borne chemicals. Different electronic noses (e-nose) [9] are being developed using gas identification systems [10] using from gas sensors. Depending on the application field, different gas sensors have been used to measure, sense, and identify different gases. MOSFET sensors, optical sensors, piezoelectric sensors (i.e., Surface Acoustic Wave, Quartz Crystal Monitor), and conductivity sensors (i.e., polymer composites, intrinsically conducting polymers, metal oxides) are some examples of the gas sensors applied in e-nose applications [11].

Although these smart gas sensors and gas identification systems are quite effective, there are still many challenges that still need to be solved [12]. One of the challenges is related to the complexity of the gas sensing principle and tasks. A particular gas sensor might be affected adversely by other gases that share common chemical properties [12]. Also, environmental factors such as humidity and temperature can impact the accuracy of the sensor. This phenomenon known as sensor drift is complex and degrades the stability of a sensor [13]. As such, the performance of e-noses and gas identification systems are adversely affected. Several factors, such as humidity, pressure, external pollution, can cause this type of instability problems, and hence, the data quality can decline over time [13]. Two main causes of the drift problem in sensors are addressed by researchers [14]. The first-order drift is related to the chemical process between the environment and the sensor. The second-order drift is directly related to the sensor noise. One approach to tackle this problem is the use of resilient sensors to drift [15].

The current state-of-the-art in sensor drift and sensor failure compensation research is the application of machine learning techniques that are widely used in many other application domains [13]. The main benefit of these techniques is that there is no need to re-calibrate the sensor. Several machine learning-based techniques have been proposed and validated to tackle this sensor drift problem for different applications, which are explained in the Related Work section. Many of these studies calibrate or correct the drift sensor values. In this study, we propose and validate a Single Plurality Voting System-based machine learning technique, which applies the majority voting rule to combine the output of the individual classifiers for tolerating the sensor failures.

Although the approach can be broadly applied, in this paper, we focus on food quality of perishable food such as beef, fish, and chicken. As a case study, we have used the beef cut quality. Within the past 50 years, the animal-based protein consumption increased to 42.20 kg per person (per year) [16], and it is estimated that beef will still be a popular animal-based protein in 2050 [17]. However, the beef quality might be affected by the potential pathogenic microorganisms, and therefore, the quality of the meat can degrade. There are several factors, such as transportation, meat chill chain, and temperature, which can affect meat quality degradation. Since microbiological methods (e.g., sensory panels, and gas chromatography) are time-consuming and require special skills, Fourier Transform Infrared (FTIR) spectroscopy and e-noses [18] have been used for meat quality control. Since the e-nose devices are cheaper, faster, and provide comparable performance to the FTIR approach, the e-nose technology fits better to beef quality monitoring. However, the drawback of the e-noses is the instability of the sensors due to several environmental conditions.

In this study, we performed our experiments by using a public dataset shared in the following link: https://dataverse.harvard.edu/dataset.xhtml?persistentId=doi:10.7910/DVN/XNFVTS. This dataset has been previously used several times by other researchers [19,20,21]; however, the power of the SPVS classification approach for this problem has not been investigated yet. In this study, we aim to analyze the applicability of SPVS classification approaches for predicting the beef cut quality. To validate our proposed approach, we performed many experiments using 11 sensors (e.g., hydrogen sulfide, ammonia, hydrogen sensors) for 12 different types of beef cuts (e.g., brisket, rib eye, tenderloin). Class labels were represented with four categories (i.e., 1-excellent, 2-good, 3-acceptable, 4-spoiled). A composed SPVS classifier has been built based on the outcome of single classifiers and using the majority voting mechanism.

The rest of this paper is organized as follows: Section 2 describes the related work. Section 3 explains the methodology. Section 4 shows our results. Section 5 presents the discussion and Section 6 provides the conclusions.

## 2. Related Work

Several techniques have been developed and validated to solve the sensor drift problem. Since our model rely on a data loss tolerable classification approach, in this section we will mainly address techniques, which were developed with machine learning approaches [12].

De Vito et al. [22] applied semi-supervised learning (SSL) methods to improve the performance of classification and regression algorithms and demonstrated that SSL approaches are effective in reducing the impact of the sensor drift and minimizing the performance degradation. Liu et al. [23] applied the domain adaptation approach for the sensor drift problem and showed that the suggested approach outperforms the traditional approaches. Yan et al. [24] proposed a new approach called maximum independence domain adaptation (MIDA) to learn domain-invariant features and, applied a semi-supervised MIDA (SMIDA) technique to solve this problem. Xue et al. [25] suggested a discrete binary version of Particle Swarm Optimization and applied in this problem. They reported that their approach is robust and does not require re-calibration. Furthermore, Component Correction-based methods [26,27,28] and Sequential Minimal Optimization-based techniques [29,30] have been applied successfully for adjusting the model to the sensor drift problem.

Zhang et al. [31] developed a framework called domain adaptation extreme learning machine and showed that this approach outperforms other drift-compensation methods. Zhao et al. [13] combined the Support Vector Machines (SVM) with the improved LSTM (Long Short-Term Memory)algorithm and demonstrated that this approach provides an accuracy of 99.0%. Vergara et al. [32] developed an ensemble technique based on Support Vector Machines (SVM) and used the weighted combination of classification algorithms that are trained at different times. Their main motivation was to identify and discriminate the six gases/analytes, namely ammonia, acetaldehyde, acetone, ethylene, ethanol, and toluene.

In addition to these approaches proposed for the sensor drift problem, there are several papers which address the prediction of beef cut quality. Wijaya [19] analyzed the stability of feature selection algorithms for sensor array optimization problem and used 12 datasets that are related to different beef cuts. They showed that a single feature selection algorithm cannot guarantee stable sensor recommendation. In this study, we performed all our experiments on the same dataset. Sarno and Wijaya [20] discussed the challenges of e-nose applications for the assessment of beef quality. Wijaya et al. [18] proposed a noise filtering framework for beef quality monitoring and showed that the framework improves the performance of multi-class classification and regression algorithms. Wijaya et al. [33] conducted several experiments and collected time series data from beef quality monitoring. Wijaya et al. [21] used K-Nearest Neighbor algorithm to classify 2/3/4 classes of beef and showed that the approach can classify fresh and spoiled beef.

According to the related work discussed in this section and to the best of our knowledge, SPVS classifiers have not been applied for meat quality prediction problem and as such, our approach has distinctive components and features for this problem. In addition, we observed that above-mentioned machine learning-based models proposed and evaluated so far (i.e., semi-supervised learning techniques, domain adaptation approach, deep learning algorithms such as LSTM) for sensor drift problem are complex and require a lot of effort to build the model.

Due to this complexity of the models such as the application of deep learning algorithms, it is also hard to explain to domain experts how the prediction is performed by the system. Since deep learning algorithms require more data compared to the traditional machine learning algorithms to build a high performance model in terms of prediction accuracy, we aimed to develop a new prediction model for beef cut quality problem by using SPVS approach. Our objective is not to achieve the highest performance, instead, we aim to develop a prediction model, which can be used in the case of sensor loss.

## 3. Methodology

**Figure 1:**
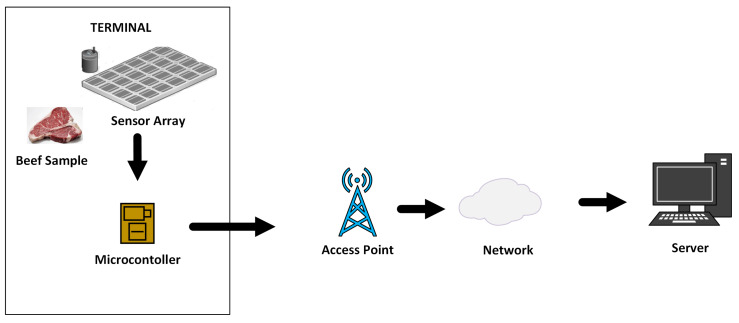
A conceptual food quality system.

The conceptual model for food quality monitoring in machine olfaction is presented in Figure 1. In such a food quality monitoring system, data acquired from the sensor array is sent to the server via the access point. The raw signals are converted into numeric values and used as input to be classified by machine learning models. Automatic determination of food freshness and quality help experts in pricing.

However, the accuracy of the sensors might be adversely affected by environmental factors such as temperature. This problem, called sensor drift, is one of the most challenging problems in chemical sensing and might cause inaccurate measurement readings and hence, impact the performance of the prediction models. There exist two kinds of sensor problems. While the first-order sensor drift is about the chemical process between the sensor and the environment, the second-order one is related to the sensor noise. In this study, we address the sensor drift problem and propose a novel model to tolerate for the sensor loss. The superiority of the proposed model is its tolerance against loss of features gathered from the sensors. In happy scenario that everything works fine individual classifiers can work with higher accuracy but in experiments that we simulate the different number of sensor losses ensemble model has higher classification accuracy.

**Figure 2:**
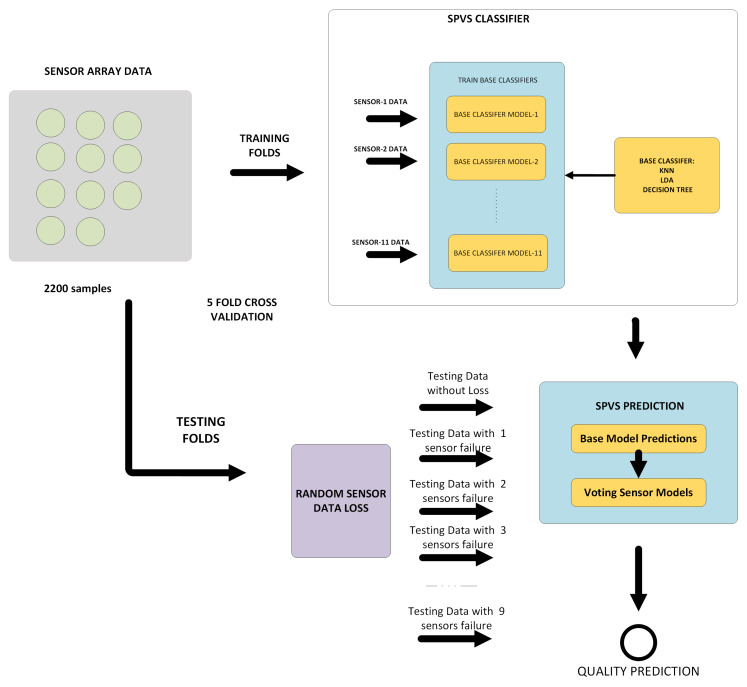
An overview of our proposed prediction approach.

The proposed method is designed to be robust against sensor failures. If a failure situation is detected and one or more sensors are ignored, system can continue automatic quality assessment. An overview of the proposed prediction approach is given in Figure 2. The sensor data is split into training and testing datasets, and then, models are trained based on each individual sensor data. 5-Fold cross-validation generalization is used in the experiments. There are 2200 samples in the data set. In each step, one fold is used as a test set, and the remaining folds are used as a training set. The training set contains no-loss data. In the test set, 10% of the data has no sensor failure, 10% of the data has one sensor failure, and the remaining of the data 2 to 9 sensor failures with the same ratio, respectively. The Ensemble SPVS model is the composition of individual classification models. Three different well-known machine learning methods are applied as base classifiers, which are LDA, DT, and kNN methods. During the prediction, outputs of the base models are combined with the majority voting, and the label with the max number of votes is considered to be the final prediction output. In the following sub-sections, we elaborate on the base classifiers that were applied as part of the SPVS-based model (Section 3.1), the adopted ensemble classifier technique and the SVP algorithm (Section 3.2), and the adopted dataset (Section 3.3).

### 3.1. Base Classifiers

During our experiments, three different classifiers are employed. These are K-Nearest Neighbor, Linear Discriminant Analysis and Decision Tree classifiers. Our first classifier, K-Nearest Neighbor, is one of the widely used distance-based algorithms, which is used for classification and regression tasks [34]. It can be considered to be one of the simplest machine learning algorithms. Unlike other complex machine learning algorithms, it has no function optimization or parameter tuning step during the training. This feature of kNN makes it not an ideal algorithm for machine learning problems with large datasets.

The fundamental principle of kNN is to search for the points, which are closest to the new data point or the data point that will be classified. K parameter represents the number of the closest neighbors from the unknown point. K parameter has a direct effect on classification results. In kNN, data points are classified based on the majority voting principle. A class label, which is most common one among its K-closest neighbors is assigned to the unlabeled data point. Different distance metrics can be used during the prediction phase. Some of the well-known distance functions are Euclidean, Manhattan, Minkowski, and Cosine functions. Definitions of these functions are presented as follows:

(1) $$\begin{array}{c} Euclidean=\sqrt{\sum_{i=1}^{n}X_{i}-Y_{i}}\\ Manhattan=\sum_{i=1}^{n}\left|X_{i}-Y_{i}\right|\\ Minkowski=(\sum_{i=1}^{n}\left(\right|X_{i}-Y_{i}{\left|\right)}^{q}{)}^{(}1/q)\\ Cosine=X\cdot Y/(||X||\times ||Y||) \end{array}$$

In Equation (1), *X* and *Y* are the different samples and $X_{i}$ and $Y_{i}$ are the feature vectors of these samples. The calculation of the first three distance metrics are basically based on simple mathematical operations. The cosine distance computation consists of vector operations such as dot and cross product. A normalization should be applied to reduce the negative effect of the features with a wide interval range. During our experiments, Euclidean distance has been used by base classifiers because this distance function is the most widely used one in machine learning applications [35].

Decision Tree (DT) classifier learns from a dataset by splitting it into different subsets. This process is repeated recursively until splitting (i.e., branching) has no effect on the prediction. This method can also be used in classification and regression. The well-known decision tree types are ID3 C4.5, and Mars algorithms. Although all these methods are very similar to each other, there are some differences for the model training. The main advantages of DT algorithms are their simplicity and speed. Its pre-processing step is shorter and simpler compared to the other alternative techniques. It can be used in both numerical and categorical data. Due to its simplicity and fast processing capability, it can process a large amount of data in a short time and this makes it preferable for problems with large datasets.

LDA is our final base classifier, which is employed in ensemble classifier constructions. LDA is originally used for dimension reduction [36]. The main purpose of the LDA is to prevent overfitting and reduce the time complexity. LDA aims to maximize the distance between classes. LDA method has five main steps. First is the computation of multi-dimensional average feature vector for all classes. The second step is the computation of the scatter matrix. The next step is the calculation of the eigen values and eigen vectors of the scatter matrix. The fourth step is the selection of the greatest eigen values. Final step is to project the original dataset into a reduced one with projection matrix W, which is obtained by using eigen values. LDA computes posteriors and, its classification mechanism relies on Bayes Theorem [37].

### 3.2. Ensemble Classifier

**Figure 3:**
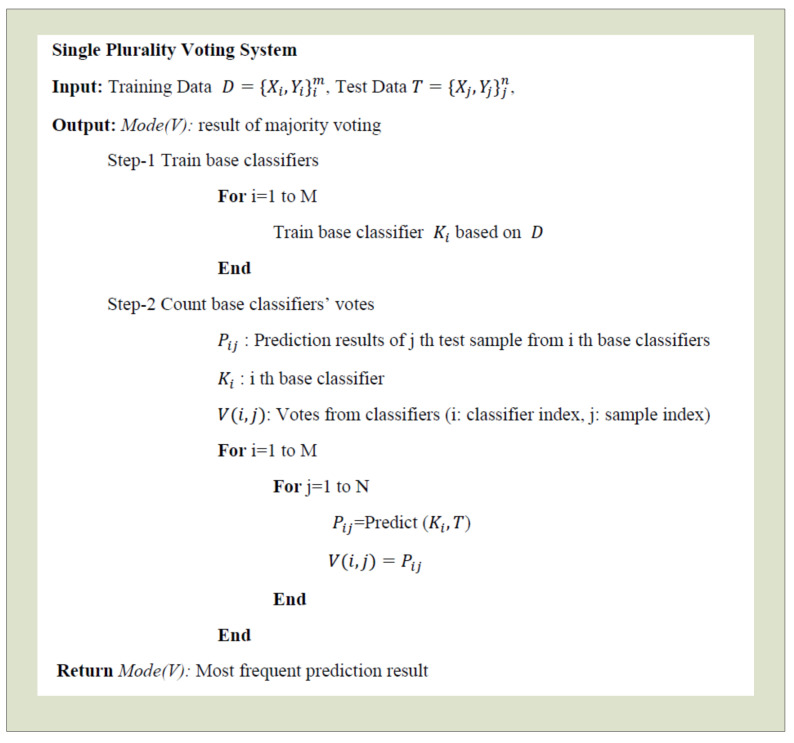
SPVS Algorithm.

SPVS is one of the approaches used in building ensemble classifiers [38]. In this method, multiple classifiers or regressors are combined and a meta-classifier is built. The meta-classifier can be either trained on the predicted class labels or probabilities from the ensemble or a majority voting can be applied to produce the final prediction. The algorithm of the SPVS classifier is shown in Figure 3. In the SPVS algorithm (Figure 3), *X* represent the feature vector of each sample, *Y* is the label of the samples, and n is the number of samples in the training set. First, several base classifiers are trained with the training dataset *D*. Each of the sample in the test set (represented as *T* in the algorithm) is classified by each base classifier individually and results are saved. In the last step, results are used by the meta-classifier to produce the final classification output. Here a secondary classifier that takes the prediction results or the probability scores of the base classifiers as input can be trained. The other ensemble technique is the majority voting, which is also applied in this study. The most frequent classification result among outputs of the base classifiers is returned as the final output value.

### 3.3. Dataset

Our experiments are performed on the publicly available time series dataset acquired with an e-nose, which is developed for beef quality monitoring experiments [39]. This dataset contains measurements from 11 different metal oxide semi-conductor gas sensors. These gas sensors and their selectivity properties are listed as follows:

- MQ135: Carbon dioxide, alcohol, ammonia, smoke, benzene
- MQ136: Hydrogen sulfide
- MQ137: Ammonia
- MQ138: Toluene, acetone, alcohol, hydrogen
- MQ2: Alcohol, hydrogen, smoke, Liquefied petroleum gas (LPG), methane, i-butane, propane
- MQ3: Methane
- MQ4: Iso-butane, propane, LPG
- MQ5: Propane, LPG
- MQ6: LNG, LPG, iso-butane, propane
- MQ8: Hydrogen
- MQ9: Carbon monoxide, methane, and propane

Measurements gathered from these sensors are recorded for 2220 min. One data point is sampled from each sensor for every minute. The dataset includes samples acquired from 12 different types of beef cuts. The type of beef cuts in the dataset are round (shank), top sirloin, tenderloin, flap meat (flank), striploin (shortloin), brisket, clod/chuck, skirt meat (plate), inside/outside, rib eye, shin, and fat. Experiments performed to create the dataset are based on the standard 2-hour method [40].

## 4. Experimental Results

During the experiments, we applied three base classifiers (kNN, LDA, and DT) for the SPVS approach. These methods are chosen because they are fast to train and test. Additionally, these methods are directly applied to the datasets. In our tables, Single Classifier methods are shown as “Single CL”, and the voting method is shown as “SPVS”. The are 12 types of beef cut meats in the dataset each with 2200 samples. For each kind, the results obtained are showed in the following figures. An additional experiment is also conducted to compare the performance of the methods when there is a failure on the measurements of sensors. In this experiment, 20% of the testing samples have no failures, 20% of the testing samples have one sensor failure, 20% of the samples have two, 20% of the samples have three and 20% of the samples have four sensor failures. The order of the failure counts, and the sensors are chosen randomly. In tables and figures, results obtained from missing values are shown with the “Missing” tag.

An additional experiment is also conducted to compare the performance of the methods when there is a failure on the measurements of the sensors. In the experiment, 10% of the testing samples have 0 failures, 10% of the testing samples have 1 sensor failure, 10% of the samples have 2, 10% of the samples have 3, 10% of the samples have 4, 10% of the samples have 5, 10% of the samples have 6, 10% of the samples have 7, 10% of the samples have 8, and 10% of the samples have 9 sensor failures. The order of the failure counts, and the sensors are chosen randomly. In the tables and figures, results obtained from this experiment are shown with the “Missing” tag.

**Figure 4:**
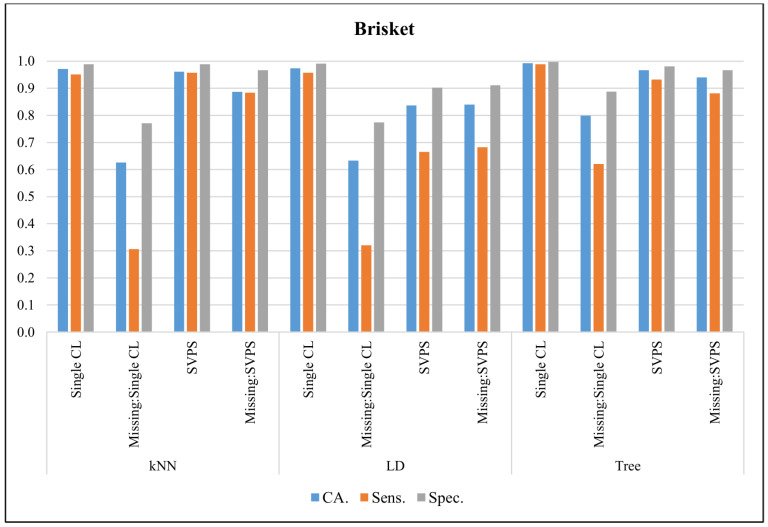
Classification results for Brisket dataset.

In Figure 4, the classification results with the Brisket dataset are presented. The Single Tree classifier provided the best CA with 99.2%, the best sensitivity with 98.8%, and the best specificity with a 99.7% score, on the dataset without missing values. The Tree-based SPVS method provided the best classification accuracy with a 94.1% score on the dataset with missing values. The kNN-based SPVS performed better on sensitivity and specificity with 88.3% and 96.7% scores, respectively.

**Figure 5:**
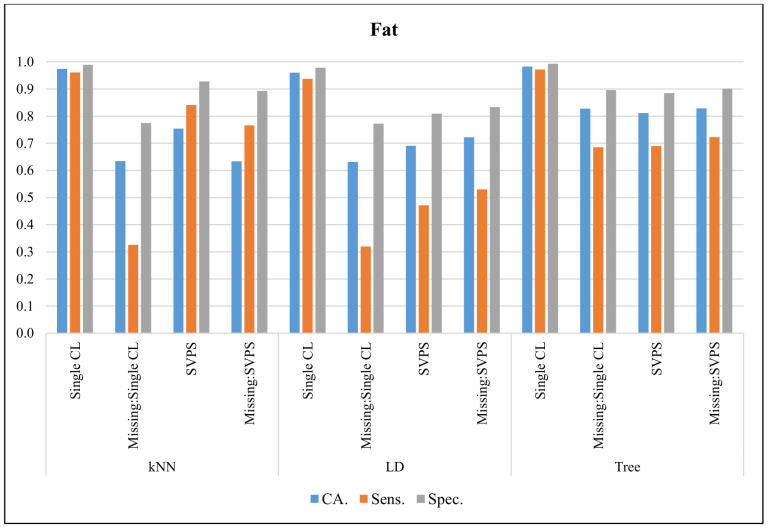
Classification results for Fat dataset.

In Figure 5, the classification results with the Fat dataset are presented. The Single Tree classifier provided the best CA with 98.3%, the best sensitivity with 97.2%, and the best specificity with a 99.3% score, on the dataset without missing values. The Tree-based SPVS method provided the best classification accuracy with an 82.8% score on the dataset with missing values. The kNN-based SPVS performed better on sensitivity and specificity with 72.3% and 90.1% scores, respectively.

**Figure 6:**
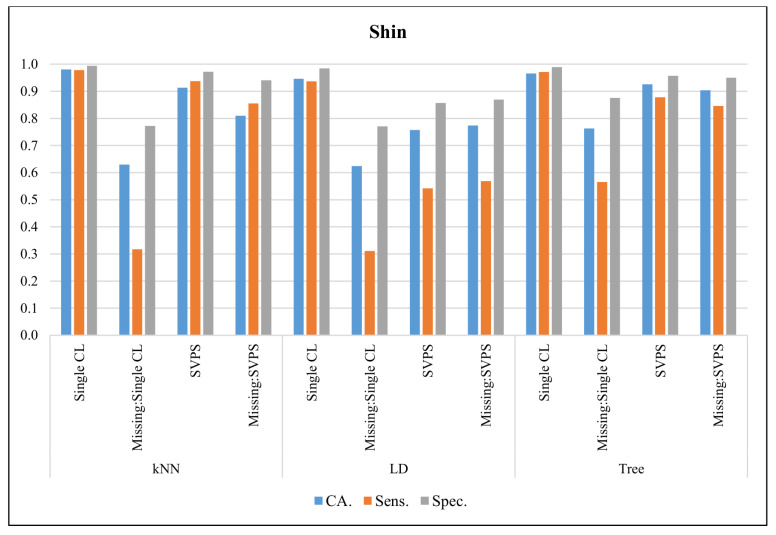
Classification results for Shin dataset.

In Figure 6, the classification results with the Shin dataset are presented. The Single kNN classifier provided the best CA with 98.1%, the best sensitivity with 97.9%, and the best specificity with a 99.3% score, on the dataset without missing values. The Tree-based SPVS method provided the best classification accuracy with a 90.4% score on the dataset with missing values. The kNN-based SPVS performed better on sensitivity and specificity with 85.5% and 94.1% scores, respectively.

**Figure 7:**
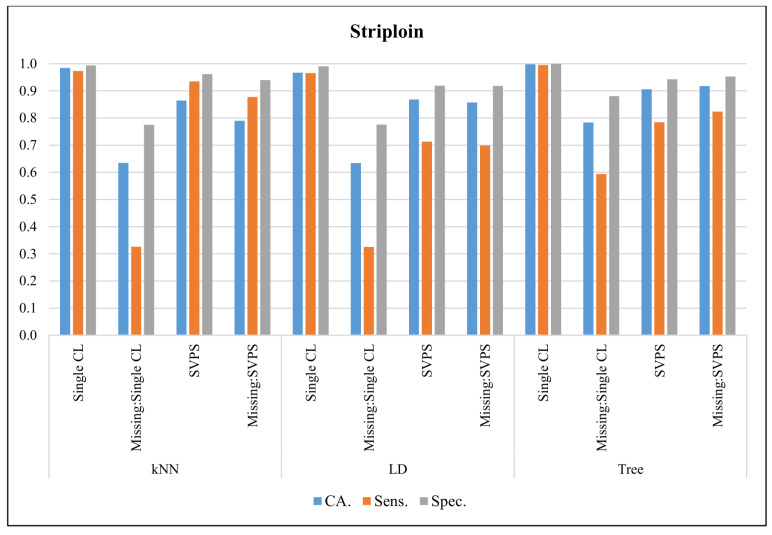
Classification results for Striploin dataset.

In Figure 7, the classification results with the Striploin dataset are presented. The Single Tree classifier provided the best classification accuracy with 99.7%, the best sensitivity with 99.5%, and the best specificity with a 99.9% score, on the dataset without missing values. The Tree-based SPVS method provided the best classification accuracy with a 91.8% score on the dataset with missing values. The kNN-based SPVS performed better on sensitivity and specificity with 87.8% and 94.0% scores, respectively.

**Figure 8:**
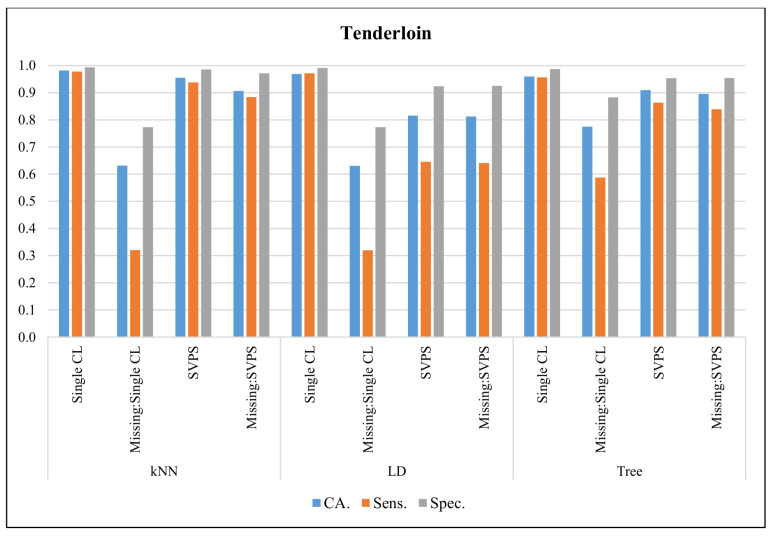
Classification results for Tenderloin dataset.

In Figure 8, the classification results with the Tenderloin dataset are presented. The kNN classifier provided the best classification accuracy with 98.2%, the best sensitivity with 97.7%, and the best specificity with a 99.4% score, on the dataset without missing values. The kNN-based SPVS method provided the best classification accuracy with a 90.7%, the best sensitivity with 88.4%, and the best specificity with a 97.1% score, on the dataset with missing values.

**Figure 9:**
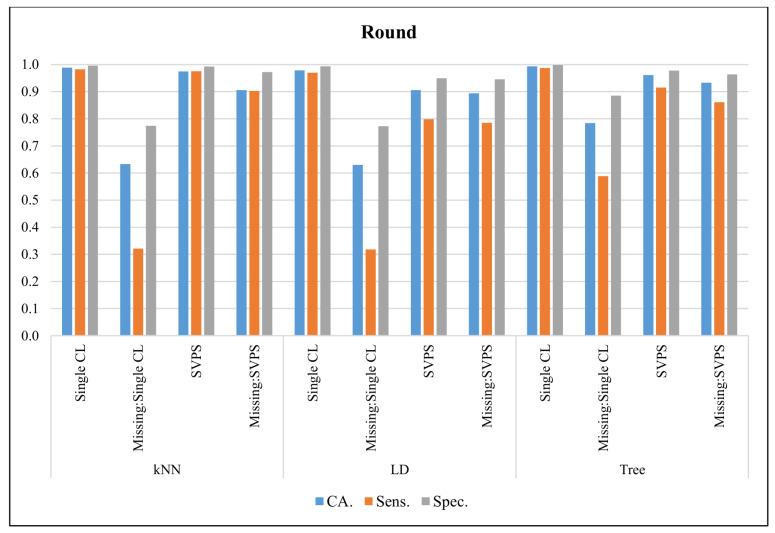
Classification results for Round dataset.

In Figure 9, the classification results with the Round dataset are presented. The Tree classifier provided the best classification accuracy with 99.3%, the best sensitivity with 98.7%, and the best specificity with a 99.8% score, on the dataset without missing values. The Tree-based SPVS method provided the best classification accuracy with a 93.3%, and the best specificity with a 96.3% score, on the dataset with missing values. The kNN-based SVPS method provided the best sensitivity with 90.3% score.

**Figure 10:**
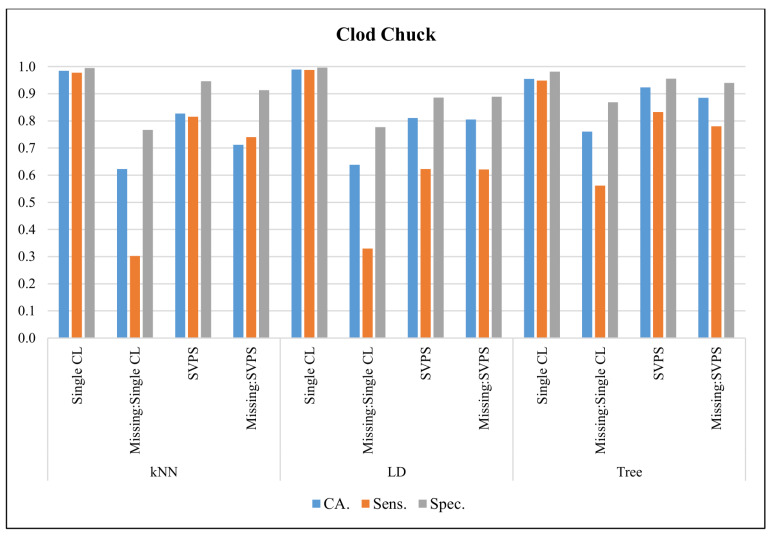
Classification results for Clod Chuck dataset.

In Figure 10, the classification results with the Clod Chuck dataset are presented. The Linear Discriminant classifier provided the best classification accuracy with 99.0%, the best sensitivity with 98.8%, and the best specificity with a 99.7% score, on the dataset without missing values. The Tree-based SPVS method provided the best classification accuracy with an 88.5%, the best sensitivity with 78.0%, and the best specificity with a 94.0% score, on the dataset with missing values.

**Figure 11:**
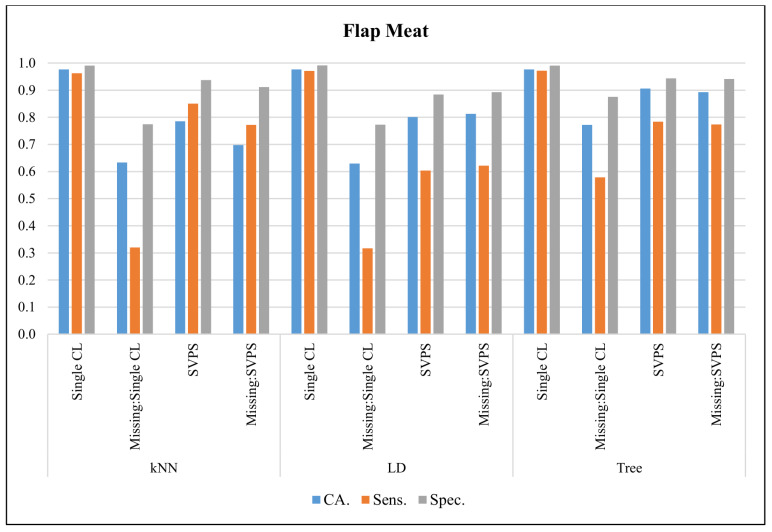
Classification results for Flap Meat dataset.

In Figure 11, the classification results with the Flap Meat dataset are presented. The Linear Discriminant classifier provided the best classification accuracy with 97.7%, the best sensitivity with 97.1%, and the best specificity with a 99.1% score, on the dataset without missing values. The kNN-based SPVS method provided the best sensitivity with a 77.2% score on the dataset with missing values. The Tree-based SPVS performed better on classification accuracy and specificity with 89.3% and 94.1% scores, respectively.

**Figure 12:**
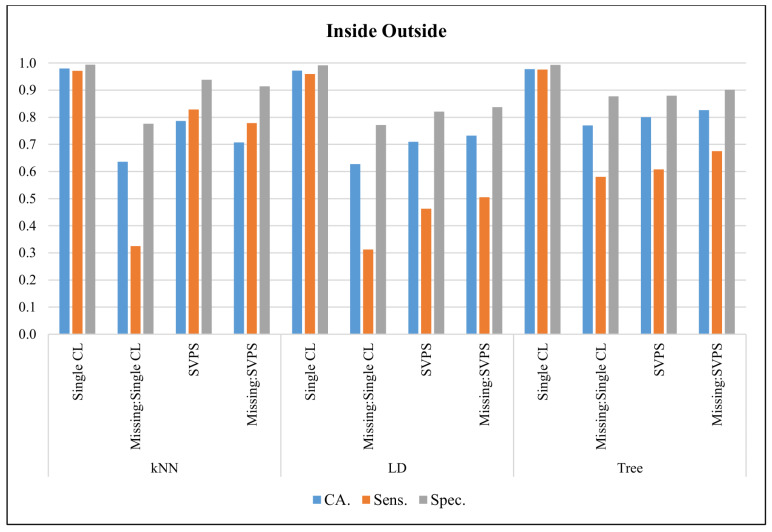
Classification results for Inside Outside dataset.

In Figure 12, the classification results with the Inside Outside dataset are presented. The Tree classifier provided the best classification accuracy with 97.8%, the best sensitivity with 97.6%, and the best specificity with a 99.3% score, on the dataset without missing values. The kNN-based SPVS method provided the best sensitivity with a 77.9% score on the dataset with missing values. The Single Tree classifier performed better on classification accuracy and specificity with 82.7% and 90.2% scores, respectively.

**Figure 13:**
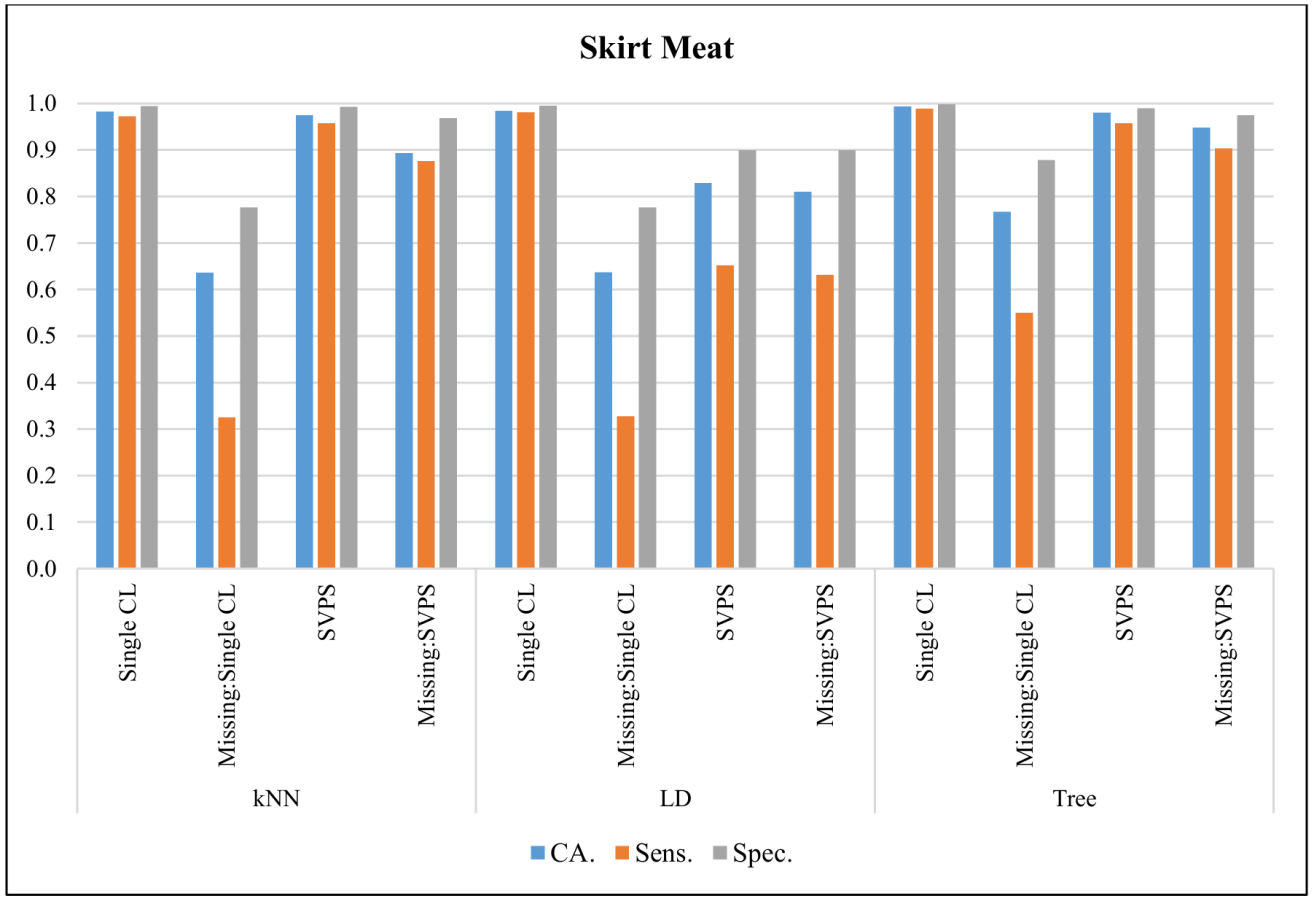
Classification results for Rib Eye dataset.

In Figure 13, the classification results with the Rib Eye dataset are presented. The Tree classifier provided the best classification accuracy with 98.7%, the best sensitivity with 97.7%, and the best specificity with a 99.6% score, on the dataset without missing values. The Tree-based SPVS method provided the best classification accuracy with a 95.3%, and the best specificity with a 97.8% score, on the dataset with missing values. The kNN-based SPVS method provided the best sensitivity with 92.4%.

**Figure 14:**
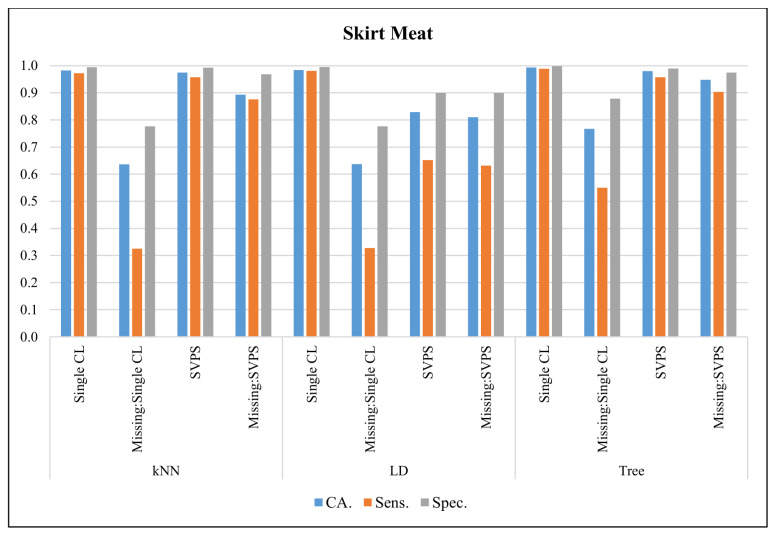
Classification results for Skirt Meat dataset.

In Figure 14, the classification results with the Skirt Meat dataset are presented. The Tree classifier provided the best classification accuracy with 99.3%, the best sensitivity with 98.8%, and the best specificity with a 99.8% score, on the dataset without missing values. The Tree-based SPVS method provided the best classification accuracy with a 94.8%, the best sensitivity with 90.3%, and the best specificity with a 97.5% score, on the dataset with missing values.

**Figure 15:**
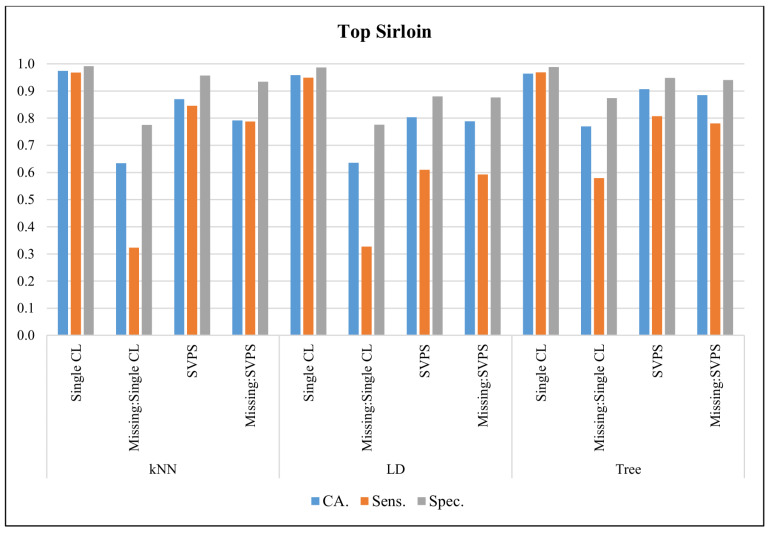
Classification results for Top Sirloin dataset.

In Figure 15, the classification results with the Top Sirloin dataset are presented. The kNN classifier provided the best classification accuracy with 97.4%, the best sensitivity with 96.8%, and the best specificity with a 99.1% score, on the dataset without missing values. The kNN-based SPVS method provided the best sensitivity with an 84.6% score on the dataset with missing values. The Tree-based SPVS classifier performed better on classification accuracy and specificity with 88.5% and 94.0% scores, respectively.

Average classification scores for methods are presented in Table 1. All the single classifiers provided better classification outcomes than the SPVS methods with different base classifiers on the datasets without missing values. However, the classification performance of these classifiers reduced significantly when it comes to tests on datasets including missing values. SPVS method’s performance is more stabilized in both experiments. The tree-based SPVS method performed better on dataset with missing values compared to all classification methods. Likewise, tree classifier is the most resistant single classifier to missing values. Linear Discriminant and kNN classifiers both performed poor with missing values.

## 5. Discussion

The interpretation of our results shows that the SPVS classifier is the most tolerable one compared to the other classifiers in the case of missing values. Although some base classifiers can reach a higher classification accuracy in the scenario where all features are used in training, this case is not possible at all times and not feasible for rapidly changing IoT environments. Sensors can be out of service due to external effects, or incorrect data might be received from the sensor at a particular time due to several problems. The proposed method presents a solution to this problem, and it was shown that the approach is efficient and effective. There are also some classifiers with feature loss tolerance such as Random Forest; however, the learning and prediction computational complexity of those classifiers is much higher.

Our main contributions in this study are listed as follows:

- An automated method for predicting the beef cut quality is proposed and validated. To the best of our knowledge, this approach is the only one that uses SPVS classifiers to support food quality prediction.
- We presented a feature loss tolerable ensemble classifier for food quality prediction.
- Several base classifiers, namely kNN, DT, and LDA, in conjunction with SPVS method are employed and analyzed for food quality prediction problem.
- It was shown that the presented approach provides better performance in the case of missing values in this problem.

Although we have used the case study for beef cut quality, the approach can be generally applied for food quality assessment. As in other experimental studies, there are some threats to the validity of this study. First, our results are based on a particular data source. This means that our results might change if applied to a different data source. However, the production of this kind of datasets is not easy, and unfortunately, not public in many cases. Due to the lack of publicly available datasets on this topic in the literature, we applied our techniques on these public datasets. Another threat is related to the number of base classifiers. Three different base classifiers have been employed; however, other researchers might perform new experiments with different classifiers because there are many algorithms in machine learning to investigate. Complementary studies could thus be used and further needed to strengthen the claims of our study. Another threat is the use of distance metrics. We adopted the Euclidean distance for the base classifiers because it is the most preferred approach. However, there are different types of distance metrics that might be used and in that case, results might be slightly different.

## 6. Conclusions and Future Work

Machine olfaction is based on automated sensing of the smell of the food using e-nose, and with this, we assess the quality of food. Many different sensors are used to detect compounds, and the proper assessment of the food quality is thus based on the correct functioning of the adopted sensors. When sensors fail to provide the correct measures, the assessment, as such, will not be reliable. To tolerate this sensor failure, we have proposed a machine learning-based approach that is based on the output of single classifiers and the majority voting mechanism. As a case study, we have adopted the case study for predicting the beef cut quality and have used the corresponding public dataset. Eleven sensors have been used during the experiments, which were performed on 12 different types of beef cuts. The automatic prediction of food quality is important to price determination because food freshness has a direct effect on the market price. Problems regarding the sensors have been simulated during our experiments, and the performance of the proposed technique has been evaluated in these cases. We showed that the presented technique provides remarkable results in the case of data loss or data quality degradation due to the sensor drift or other types of failures. As we demonstrated, ensemble learning approaches have a huge potential to tolerate data loss and to predict the food quality accurately.

In our future work, we will investigate different ensemble learning approaches with various settings to improve the performance and extend our experimental results with more datasets.

## Figures and Tables

**Table 1:** Average classification scores for classifiers.

Base Classifier	Method	CA	Sens	Spec
**kNN**	Single CL	0.9795	0.9694	0.9926
	Missing:Single CL	0.6700	0.3918	0.7976
	SVPS	0.8869	0.9039	0.9659
	Missing:SVPS	0.8680	0.8884	0.9604
**LD**	Single CL	0.9705	0.9630	0.9903
	Missing:Single CL	0.6714	0.3954	0.7991
	SVPS	0.8073	0.6244	0.8880
	Missing:SVPS	0.8102	0.6295	0.8903
**Tree**	Single CL	0.9800	0.9765	0.9931
	Missing:Single CL	0.8703	0.7626	0.9354
	SVPS	0.9113	0.8255	0.9482
	Missing:SVPS	0.9071	0.8182	0.9463

## Abbreviations

The following abbreviations are used in this manuscript:

IoT	Internet of Things
kNN	K-Nearest Neighbor
LDA	Linear Discriminant Analysis
MIDA	maximum independence domain adaptation
SPVS	Single Plurality Voting System
SSL	Semi-Supervised Learning

## References

[1] Guthrie B. (2017). Machine Olfaction. Springer Handbook of Odor.

[2] Gardner J.W., Persaud K.C., Gouma P., Gutierrez-Osuna R. (2012). Guest Editorial—Special issue on machine olfaction. IEEE Sens. J..

[3] Chen J., Gu J., Zhang R., Mao Y., Tian S. (2019). Freshness evaluation of three kinds of meats based on the electronic nose. Sensors.

[4] Liu L., Li X., Li Z., Shi Y. (2018). Application of Electronic Nose in Detection of Fresh Vegetables Freezing Time Considering Odor Identification Technology. Chem. Eng. Trans..

[5] Stassen I., Bueken B., Reinsch H., Oudenhoven J., Wouters D., Hajek J., Van Speybroeck V., Stock N., Vereecken P., Van Schaijk R. (2016). Towards metal–organic framework based field effect chemical sensors: UiO-66-NH 2 for nerve agent detection. Chem. Sci..

[6] Van Geffen W.H., Bruins M., Kerstjens H.A. (2016). Diagnosing viral and bacterial respiratory infections in acute COPD exacerbations by an electronic nose: A pilot study. J. Breath Res..

[7] Eamsa-Ard T., Seesaard T., Kitiyakara T., Kerdcharoen T. Screening and discrimination of Hepatocellular carcinoma patients by testing exhaled breath with smart devices using composite polymer/carbon nanotube gas sensors. Proceedings of the 2016 9th Biomedical Engineering International Conference (BMEiCON).

[8] Wilson A.D. (2018). Application of electronic-nose technologies and VOC-biomarkers for the noninvasive early diagnosis of gastrointestinal diseases. Sensors.

[9] Wilson A.D., Baietto M. (2009). Applications and advances in electronic-nose technologies. Sensors.

[10] Bang Y.K., Lee C.H. (2018). Design of a Hierarchically Structured Gas Identification System Using Fuzzy Sets and Rough Sets. Trans. Korean Inst. Electr. Eng..

[11] Arshak K., Moore E., Lyons G.M., Harris J., Clifford S. (2004). A review of gas sensors employed in electronic nose applications. Sens. Rev..

[12] Feng S., Farha F., Li Q., Wan Y., Xu Y., Zhang T., Ning H. (2019). Review on Smart Gas Sensing Technology. Sensors.

[13] Zhao X., Li P., Xiao K., Meng X., Han L., Yu C. (2019). Sensor Drift Compensation Based on the Improved LSTM and SVM Multi-Class Ensemble Learning Models. Sensors.

[14] Ma Z., Luo G., Qin K., Wang N., Niu W. (2018). Online sensor drift compensation for E-nose systems using domain adaptation and extreme learning machine. Sensors.

[15] Sunil T., Chaudhuri S., Mishra V. (2015). Optimal selection of SAW sensors for E-Nose applications. Sens. Actuators Chem..

[16] Sans P., Combris P. (2015). World meat consumption patterns: An overview of the last fifty years (1961–2011). Meat Sci..

[17] Bruinsma J. (2003). World Agriculture: Towards 2015/2030: An FAO Perspective.

[18] Wijaya D.R., Sarno R., Zulaika E. (2019). Noise filtering framework for electronic nose signals: An application for beef quality monitoring. Comput. Electron. Agric..

[19] Wijaya D.R., Afianti F. (2020). Stability Assessment of Feature Selection Algorithms on Homogeneous Datasets: A Study for Sensor Array Optimization Problem. IEEE Access.

[20] Sarno R., Wijaya D.R. (2019). Recent development in electronic nose data processing for beef quality assessment. Telkomnika.

[21] Wijaya D.R., Sarno R., Zulaika E., Sabila S.I. (2017). Development of mobile electronic nose for beef quality monitoring. Procedia Comput. Sci..

[22] De Vito S., Fattoruso G., Pardo M., Tortorella F., Di Francia G. (2012). Semi-supervised learning techniques in artificial olfaction: A novel approach to classification problems and drift counteraction. IEEE Sens. J..

[23] Liu Q., Li X., Ye M., Ge S.S., Du X. (2013). Drift compensation for electronic nose by semi-supervised domain adaption. IEEE Sens. J..

[24] Yan K., Kou L., Zhang D. (2017). Learning domain-invariant subspace using domain features and independence maximization. IEEE Trans. Cybern..

[25] Ur Rehman A., Bermak A. (2018). Heuristic random forests (HRF) for drift compensation in electronic nose applications. IEEE Sens. J..

[26] Ziyatdinov A., Chaudry A., Persaud K., Caminal P., Perera A. (2009). Common principal component analysis for drift compensation of gas sensor array data. AIP Conf. Proc..

[27] Padilla M., Perera A., Montoliu I., Chaudry A., Persaud K., Marco S. (2010). Drift compensation of gas sensor array data by orthogonal signal correction. Chemom. Intell. Lab. Syst..

[28] Artursson T., Eklöv T., Lundström I., Mårtensson P., Sjöström M., Holmberg M. (2000). Drift correction for gas sensors using multivariate methods. J. Chemom..

[29] Gong J.W., Chen Q.F., Lian M.R., Liu N.C., Daoust C. (2006). Temperature feedback control for improving the stability of a semiconductor-metal-oxide (SMO) gas sensor. IEEE Sens. J..

[30] Rebholz J., Weimar U., Barsan N. (2014). Influence of conduction mechanism changes on the sensor performance of SMOX based gas sensors. Procedia Eng..

[31] Zhang L., Zhang D. (2014). Domain adaptation extreme learning machines for drift compensation in E-nose systems. IEEE Trans. Instrum. Meas..

[32] Vergara A., Vembu S., Ayhan T., Ryan M.A., Homer M.L., Huerta R. (2012). Chemical gas sensor drift compensation using classifier ensembles. Sens. Actuators Chem..

[33] Wijaya D.R., Sarno R., Zulaika E. (2018). Electronic nose dataset for beef quality monitoring in uncontrolled ambient conditions. Data Brief.

[34] Duda R.O., Hart P.E., Stork D.G. (2001). Pattern Classification.

[35] Deza M.M., Deza E. (2006). Dictionary of Distances.

[36] McLachlan G.J. (2004). Discriminant Analysis and Statistical Pattern Recognition.

[37] Klett J. (1972). Applied Multivariate Analysis.

[38] Dangeti P. (2017). Statistics for Machine Learning.

[39] Wijaya D.R. (2019). Dataset for Electronic Nose from Various Beef Cuts. https://ieee-dataport.org/documents/dataset-electronic-nose-various-beef-cuts.

[40] Prescott L.M., Harley J.P., Klein D.A. (2005). Microbiology.

